# Association between Influenza and COVID-19 Viruses and the Risk of Atherosclerosis: Meta-Analysis Study and Systematic Review

**DOI:** 10.3390/arm90040043

**Published:** 2022-08-12

**Authors:** Mahsa Jalili, Kourosh Sayehmiri, Nastaran Ansari, Behzad Pourhossein, Maryam Fazeli, Farid Azizi Jalilian

**Affiliations:** 1 Department of Virology, Faculty of Medicine, Hamadan University of Medical Sciences, Hamadan 6517838738, Iran; 2 Department of Microbiology, Faculty of Medicine, Hamadan University of Medical Sciences, Hamadan 6517838636, Iran; 3 Department of Biostatistics, School of Health, Ilam University of Medical Sciences, Ilam 6931851147, Iran

**Keywords:** influenza virus, COVID-19, SARS-CoV-2, 2019-nCOV, atherosclerosis, meta-analysis

## Abstract

**Highlights:**

- Atherosclerosis is an important issue in health care systems and one of the main causes of morbidity and mortality in patients.
- There is evidence of an association between infectious diseases and atherosclerosis.
- The present study demonstrates that infections with influenza and COVID-19 are risk factors for arthrosclerosis.
- It seems necessary to consider different strategies for managing and eradicating viral infections.

**Abstract:**

There is a lot of evidence to suggest that patients infected with the COVID-19 and influenza viruses are at risk of atherosclerosis. Additionally, there are heterogeneous studies on the risk of arthrosclerosis in patients infected with the influenza and COVID-19 viruses. We conducted a case–control and cross-sectional study and examined the association between the risk of atherosclerosis, and *influenza virus (IV-A and IV-B) and* *COVID-19* infections in this study. We searched for keywords such as influenza virus, COVID-19 and atherosclerosis in English and Persian in well-known databases such as PubMed, SID, Magiran and Google Scholar. In this study, we analyzed the information using a meta-analysis, the random effect model, the I2 index and STAT (version 11.2). The results from the analysis of ten studies on *influenza virus* and nine studies on *COVID-19* reviewed individually (totaling 6428 samples for *influenza virus* infections and 10,785 samples for *COVID-19* infections) demonstrated a risk of arthrosclerosis in patients with influenza and COVID-19 infections, with an OR (odds ratio) = 0.45 ((95% CI): 0.25 to 0.64) and an OR (odds ratio) = 1.04 ((95% CI): 0.82 to 1.26), respectively. The present study provides new insights into the risk of atherosclerosis in patients infected with the COVID-19 and influenza viruses. Therefore, it seems necessary to consider different strategies for managing and eradicating viral infections among individuals.

## 1. Introduction

Preventive health care has been proposed to reduce the heavy burden of health care in communities worldwide [1]. Arthrosclerosis is one of the leading causes of morbidity and mortality in older adults in developed countries [2]. Furthermore, many efforts have been made to correct or treat atherosclerosis and its complications. Additionally, atherosclerosis, as an inflammatory disease, affects the walls of blood vessels. This disease is characterized by the accumulation and progression of lipids in the walls of the arteries [3]. Furthermore, many factors cause atherosclerosis, including smoking, high blood pressure, many diseases and other environmental factors [4]. 

Many seroepidemiological studies have shown that persistent infections with bacterial or viral agents may be associated with atherosclerosis [5]. Evidence suggests that human atherosclerosis is associated with previous exposure to some pathogens such as *Chlamydia pneumoniae*, *Helicobacter pylori*, HIV, *Porphyromonas gingivalis*, CMV, Herpes simplex virus types 1 and 2, Entro viruses, hepatitis A and *influenza virus* [6].

In a recent outbreak of *COVID-19* infections, significant mortality was attributed to heart disease and atherosclerosis [7]. Previous studies have demonstrated conflicting reports on the association between infections with the *influenza virus (IV)* and *coronavirus*, and arthrosclerosis [8]. The results of some studies have demonstrated that *influenza virus* (*IV-A* and *IV-B*) and *COVID-19* infections cannot predict the risk of arthrosclerosis and that the infectious burdens of these diseases are independent [9]. On the other hand, some researchers suggested that *influenza virus* (*IV-A* and *IV-B*) and *COVID-19* infections may play roles in the development of atherosclerosis by specific mechanisms [10].

Due to the recent *influenza virus* (*IV-A* and *IV-B*) and *COVID-19* epidemics, and the acute condition of patients with atherosclerosis, we hypothesized that previous *COVID-19* and *influenza virus* (*IV-A* and *IV-B*) infections were associated with atherosclerosis. To prove this hypothesis, we conducted a meta-analysis study and systematic review study to investigate the association between *influenza virus* (*IV-A* and *IV-B*) and *COVID-19* infections, and atherosclerosis. 

## 2. Methods

### 2.1. Study Instructions

The present study was conducted according to the PRISMA guidelines [11]. The study steps included searching for articles, selecting articles, evaluating the quality of the studies, extracting article information and performing statistical analyses. All steps were performed independently by two different authors. The third author supervised the collection of studies and acted as a judge for disagreements.

### 2.2. Eligibility Criteria and Information Sources

In order to identify appropriate studies on the risk of atherosclerosis in patients with COVID-19 and influenza, we considered case–control and cross-sectional studies. In this study, to avoid bias, all steps in the collection of studies were performed by two researchers. The third researcher only stepped in to further monitor and manage the process.

### 2.3. Search Strategy

We searched well-known databases. At first, reputable databases containing medical information were searched until 16 August 2020. These databases included ISI, Pubmed, EMBASE, MEDLINE, Cochrane and Google scholar. In this study, the search was performed based on the following keywords: “influenza virus” [MeSH], “coronavirus” [MeSH], “COVID-19” [MeSH] and “atherosclerosis”.

### 2.4. Quality of Evaluation

In the present study, the Newcastle–Ottawa Scale (NOS) was used to evaluate non-standard studies. Finally, according to this checklist, the two researchers rated each of studies. To evaluate the quality, “high”, “medium” or “low” quality were considered for the articles. Hence, for each study, a score of more than 7 was considered to be of “high quality”, a score of 4–6 was considered to be of “average quality”, and a score of less than 4 was considered to be of “low quality”.

### 2.5. Included and Excluded Studies

In the present study, the inclusion criteria for CC studies (case–control studies) and CS study (cross-sectional studies) included influenza and coronavirus infections in patients with atherosclerosis.

The exclusion criteria include (1) studies that did not focus on influenza and coronavirus infections in patients with atherosclerosis, (2) duplicate studies, (3) studies with the full text not written in English, (4) studies for which the full text was inaccessible, and (5) studies that were not relevant to this topic. 

### 2.6. Study Selection

In this study, all studies were imported into Zotero software (5.0.21; GMU, CHNM, Fairfax, VA, USA), and duplicate studies were eventually removed after “Finding Updates”. After deleting the authors names, journal names, and year of publication for all studies, each study was evaluated independently by the authors. In this study, all articles were screened separately by two authors (articles were reviewed by reading the title and summary of the article, as well as the titles and criteria for entry and exit of all articles (eligibility stage)). An expert researcher also participated in the final selection of articles, and the final decision was made by the expert researcher.

### 2.7. Information Extraction

In summary, an appropriate checklist was considered for the present study. The checklist included the author name(s), year of publication, place of study, sample size, tracking time, relative risk (RR), odds ratio (OR) with 95% CI (confidence interval), number of events in both groups (case and control) and age of participants. Additionally, the data were extracted by two researchers separately. The third researcher managed and monitored the information. 

### 2.8. Statistical Analysis

In the present study, two tests were used to evaluate the articles: these two tests included Cochran’s Q test and the I2 index. The I2 index consists of four categories. A description of the classification of this index is described in Reference [12]. To obtain heterogeneity in articles, the subgroup analysis method was used. To measure the predictive power of each article, the sensitivity analysis method was used. To measure publication bias in the articles, Funnel design tests, and Egger and Begg tests were used. Finally, to obtain the statistical results, Version 2 of the meta-analysis software including: meta version 4.9-1 with R software version 3.5.1 was used. A value of *p* < 0.05 was considered for the evaluation of the tests. 

## 3. Results

### 3.1. Search for Studies

**Figure 1:**
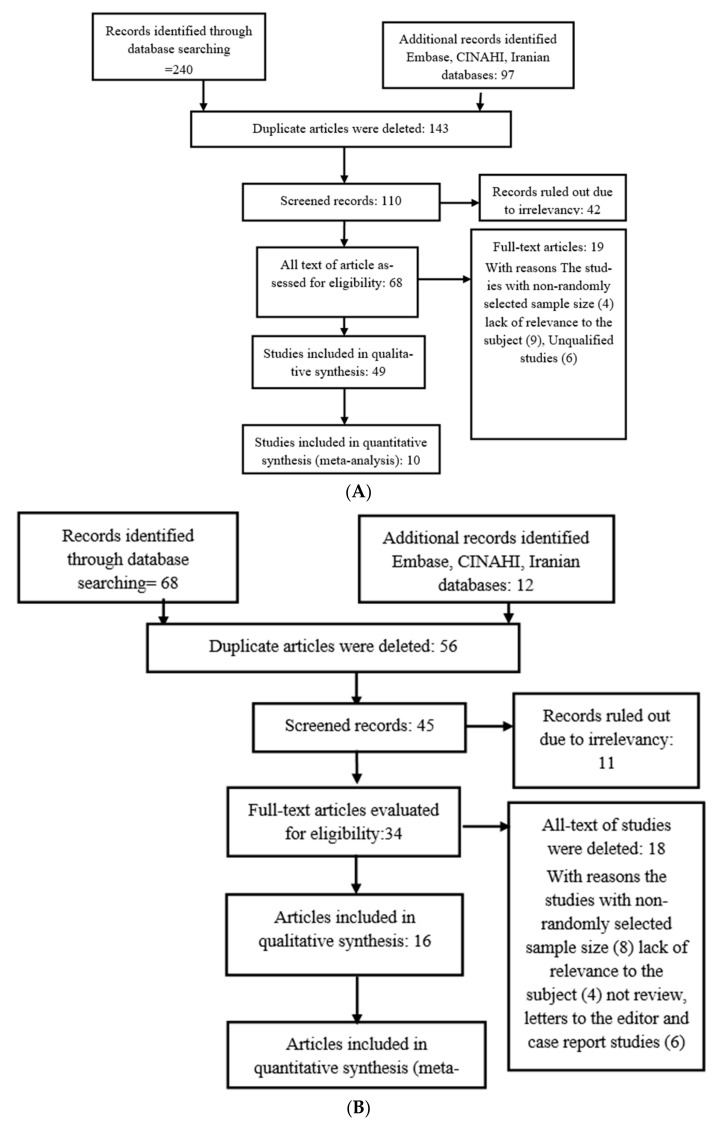
Flowchart of the selection studies for the analysis of influenza virus and its association with atherosclerosis (**A**). Flowchart of the selection studies for the analysis of COVID-19 (coronavirus) and its association with atherosclerosis (**B**).

A total of *N* = 287 studies were obtained from well-known databases and evaluated by two authors; the authors manually identified *N* = 21 articles (a total of 240 articles on influenza viruses and 68 articles on COVID-19 infection), and *N* = 199 (143 articles on influenza viruses and 56 articles on COVID-19 infection) articles were ruled out due to duplication. After screening the title and abstracts, *N* = 155 (110 articles on influenza viruses and 45 articles on COVID-19 infection) articles were ruled out due to irrelevancy. After finding the full text of an article, *N* = 102 (68 articles on influenza viruses and 34 articles on COVID-19 infection) articles were removed because their focus was not on our topic of interest. Additionally, articles with the full text not written in English or with inaccessible full texts (*N* = 37) (19 articles on influenza viruses and 18 articles on COVID-19 infection), letters to the editor without original information and true data (*N* = 12) (6 articles on influenza viruses and 6 articles on COVID-19 infection), review articles and case reports (*N* = 39) (25 articles on influenza viruses and 3 articles on COVID-19 infection), and articles of a low quality (*N* = 7) (14 articles on influenza viruses and 4 articles on COVID-19 infection) were removed. Finally, *N* =19 (10 articles on influenza viruses and 9 articles on COVID-19 infection) articles of good quality were included in this study (Figure 1).

### 3.2. Article Exclusivities 

Relevant articles about atherosclerosis published in English were considered per the inclusion criteria. Articles with a selected non-random sample size, articles not related to the topic of interest, letters to the editor and case reports were removed (as explained in more detail above).

### 3.3. Risk of Bias in Articles

In the present study, articles of all methodological types were reviewed and then selected. Additionally, papers were evaluated according to their method and needed to receive sufficient points before inclusion in the present study.

### 3.4. Outcome of Articles

In an initial search performed by two independent researchers, the present study searched for related articles (*N* = 287) in the various databases mentioned above. The final articles consisted of CC studies (case–control studies) and CS studies (cross-sectional studies) that individuals met the criteria for entering meta-analysis and systematic review (Figure 1A,B).

### 3.5. Evaluation of Outcome

**Figure 2:**
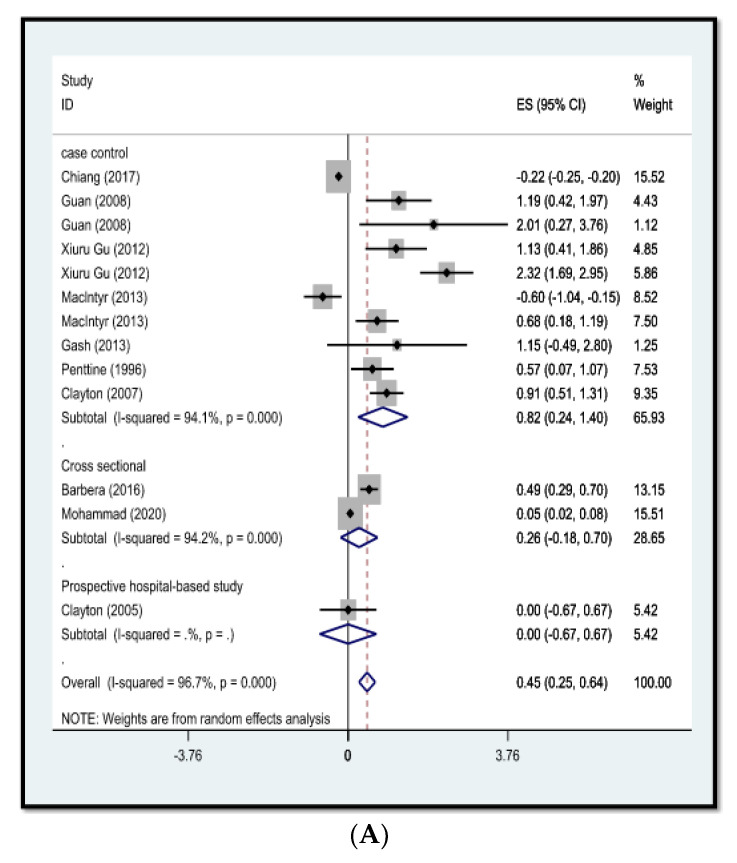

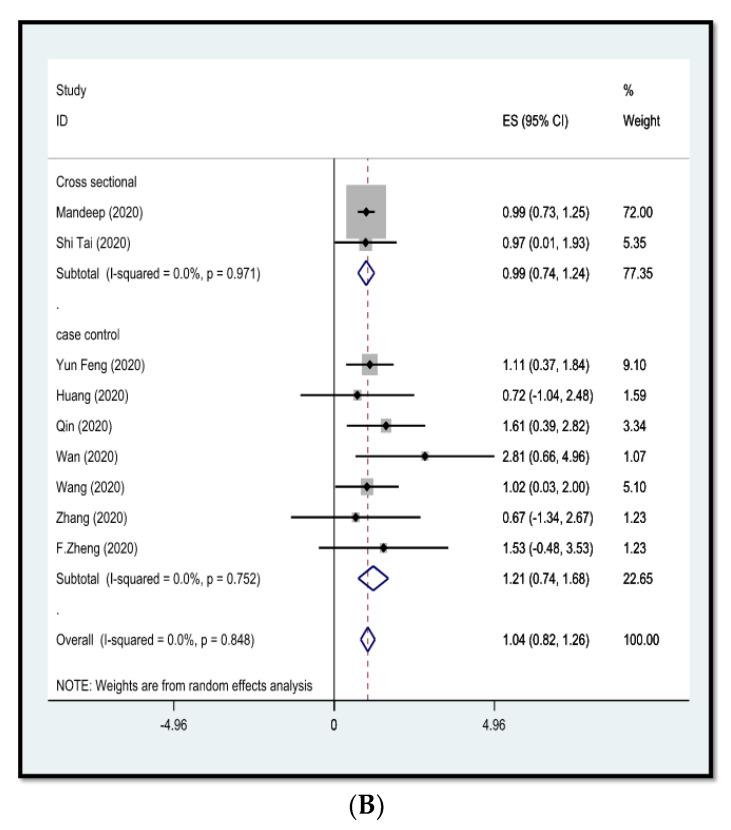
This study assessed the association between influenza virus and atherosclerosis. In this chart, the studies are categorized into case– control, cross-sectional and hospital-based studies and separated by year published and name of the authors, supported by a model of random effects (**A**); [13,14,15,16,17,18,19,20,21]. A meta-analysis was performed on the association between COVID-19 virus infection and atherosclerosis. In this chart, the studies are categorized into case–control, cross-sectional and hospital-based studies and separated by year published and name of the authors, supported by a model of random effects (**B**); [7,8,9,10,22,23,24,25,26]. PRISMA model in meta-analysis studies [11].

From a statistical point of view, an estimation of the association between influenza virus and COVID-19 (coronavirus) infections, and atherosclerosis is critical, and in the present study, we obtained OR (odds ratio) = 0.45 (95%CI: 0.25 to 0.64) and OR (odds ratio) = 1.04 (95%CI: 0.82 to 1.26), respectively (Figure 2).

### 3.6. Additional Outcome

**Figure 3:**
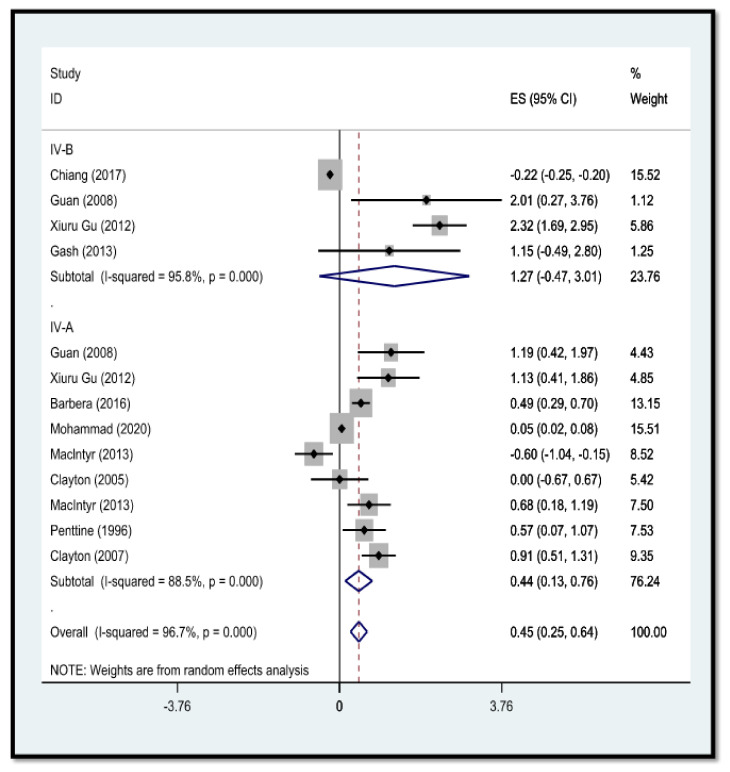
A meta-analysis of the relationship between influenza virus types (types A and B) and atherosclerosis was performed. Throughout this chart, the studies are separated based on the types of influenza virus and their associations with atherosclerosis, supported by a random effects model; [13,14,15,16,17,18,19,20,21]. PRISMA model in meta-analysis studies [11].

**Figure 4:**
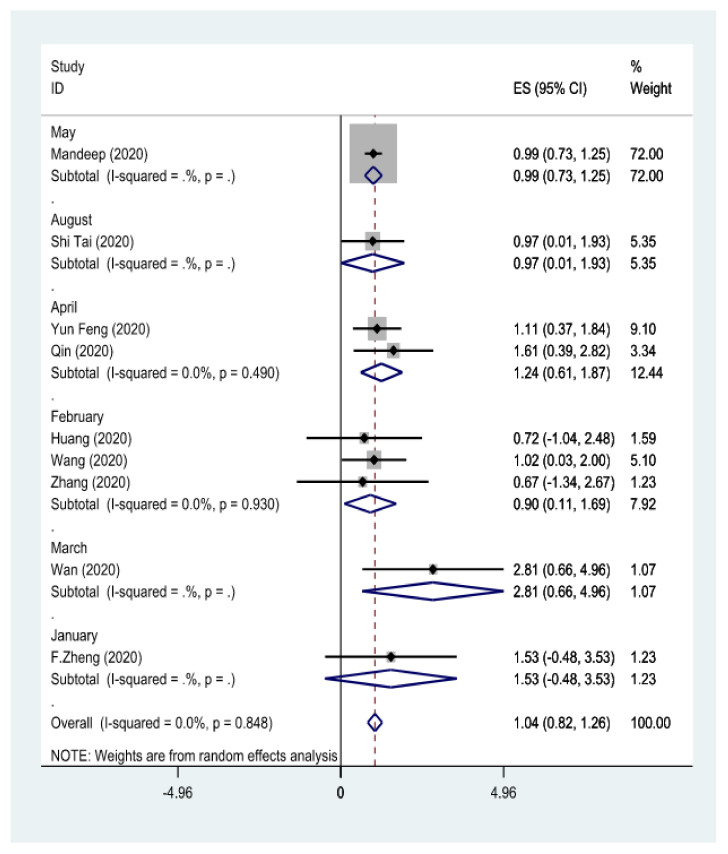
The correlation between a coronavirus or COVID_19 infection and atherosclerosis was assessed. Throughout this chart, the studies are separated based on the months of 2020, supported by a random effects model conducted by various authors; [8,9,10,22,23,24,25,26]. PRISMA model in meta-analysis studies [11].

In the present study, a qualitative evaluation was performed. A quality rating was given to these studies (Table 1 and Table 2). In the present study, articles on the relationship between *influenza virus* infection and arthrosclerosis were separated for different types of *influenza virus* (*types IV-A and IV-B*) in separate flowcharts (Figure 3). Additionally, in another flowchart, the relationship between *coronavirus* infection and arthrosclerosis was separated based on the months of the year 2020 (Figure 4).

## 4. Discussion

### Consolidation of Evidence

Today, infectious diseases affect people much more severely and are spreading at a higher rate in all parts of the world. In the United States and most other developed countries, arthrosclerosis is one of the leading causes of morbidity and mortality. In 2016, atherosclerosis, which affects the arteries of the heart and brain, caused nearly 18 million deaths worldwide. Atherosclerosis, or hardening of the arteries, is a complication that causes plaque to form inside the arteries [28,29]. Plaques are made of cholesterol, fatty substances, cell waste products, calcium and fibrin (blood-clotting agent) [30]. There is evidence of an association between infectious diseases and atherosclerosis. There are studies that have suggested bacterial and viral pathogens as one of the initiators or enhancers of atherosclerosis [31]. Several seroepidemiological and immunohistochemical studies were performed on viral infections, especially on different types of *influenza viruses* and on the novel *coronavirus (COVID-19)*, with atherosclerosis [22]. Several studies demonstrated a possible association between *influenza virus* and *coronavirus* infections as a risk factor for plaque rupture (thrombosis) and infarction [23]. On the other hand, the effects of influenza virus and COVID-19 infections on atherosclerosis are highly controversial [24]. According to these confusing results, we decided to investigate the roles of *influenza viruses (IV-A and IV-B)* and *coronavirus* in atherosclerosis in the form of a meta-analysis study. Finally, in the present article, different types of *influenza virus (IV-A, IV-B)* and *coronavirus* were considered as two emerging risk factors in atherosclerosis. 

## 5. Conclusions

In the present article, the association among *influenza virus*, COVID-19 (*coronavirus)*, and atherosclerosis was evaluated. Briefly, from the results of this study, there is a relationship between the presence of *influenza virus* and *coronavirus* infections, and atherosclerosis. In addition, the results of this study showed that infections with the *influenza virus* and the *coronavirus* are one of the risk factors for atherosclerosis [25]. Therefore, a new approach must be taken to manage viral infections.

## Figures and Tables

**Table 1:** Characteristics of the studies included in the meta-analysis study on the association between *influenza virus* and atherosclerosis.

ID	Ref	Writer	The Year the Study Was Conducted	The Country in Which the Study Was Conducted	Design	The Average Age of Study Participants	Effective Size			*p* Value	Number of Sample	Type of Flu	Quality
							OR	95% Cl					
1	[13]	Chiang	2017	Taiwan	CC	76.8	0.8	0.78	0.82	0.01	160	IV-B	High
2-1	[14]	Guan	2008	China	CC	60.0	3.3	1.5	7.04	0.003	209	IV-A	Moderate
2-2	[14]	Guan	2008	China	CC	60.0	7.5	1.3	43.0	0.001	209	IV-B	Moderate
3-1	[15]	Xiuru Gu	2012	China	CC	57.0	3.1	1.5	6.4	0.004	252	IV-A	High
3-2	[15]	Xiuru Gu	2012	China	CC	57.0	10.2	5.7	20.0	0.001	252	IV-B	High
4	[16]	Barbera	2016	Spain	CS	85.0	1.64	1.33	2.02	0.001	728	IV-A	Moderate
5	[17]	Mohammad	2020	Sweden	CS	75.0	1.05	1.02	1.08	0.002	562	IV-A	Moderate
6-1	[18]	Maclntyr	2013	Australi	CC	65.0	0.55	0.35	0.85	0.008	559	IV-B	Weak
6-2	[18]	MacIntyr	2013	Australi	CC	65.0	1.98	1.2	3.3	0.001	559	IV-A	Weak
7	[27]	Clayton	2005	USA	PHS	63.0	1.0	0.5	1.9	0.002	534	IV-A	High
8	[19]	Gash	2013	London	CC	40.0	3.17	0.61	16.47	0.002	137	IV-B	High
9	[20]	Penttine	1996	Finland	CC	49.5	1.77	1.07	2.93	0.001	3172	IV-A	Moderate
10	[21]	Clayton	2007	UK	CC	72.0	2.48	1.67	3.7	0.002	115	IV-A	Moderate
											*N* total: 6428		

OR: odds ratio, CC: case–control studies, CS: cross-sectional study, N and NO: number, PHS: prospective hospital-based study.

**Table 2:** Characteristics of the studies included in the meta-analysis study on the association between COVID-19 (*coronavirus*) infection and atherosclerosis.

ID	Ref	Author	Years	Months	Country	Design	Mean Age	Effect Size			*p* Value	Sample Size	Quality
								OR	95% Cl				
1	[22]	Mandeep	2020	May	Boston	CS	65	2.70	2.08	3.51	0.001	8910	Moderate
2	[23]	Shi Tai	2020	August	China	CC	51	2.65	1.01	6.89	0.001	332	High
3	[24]	Yun Feng	2020	April	China	CC	58	3.02	1.45	6.32	0.004	476	High
4	[25]	Huang	2020	February	China	CC	49	2.05	0.43	14.54	0.001	41	Moderate
5	[7]	Qin	2020	April	China	CC	61	4.98	1.48	16.79	0.002	452	Weak
6	[8]	Wan	2020	March	China	CC	56	16.59	1.93	142.0	0.002	135	Moderate
7	[9]	Wang	2020	February	China	CC	66	2.76	1.03	7.35	0.001	138	High
8	[10]	J. Zhang	2020	February	China	CC	64	1.95	0.62	34.11	0.004	140	High
9	[26]	F. Zheng	2020	January	China	CC	57	4.61	0.62	34.11	0.002	161	Moderate
												*N* total: 10,785	

OR: odds ratio, CC: case–control studies, CS: cross-sectional study, N and NO: number.

## Data Availability

All included studies were publicly available and published in indexed journals.
